# 3D Printer-Assisted Layered Fabrication of a Novel Appliance for Oral Myofunctional Therapy and Functional Evaluation of Its Effects on Orofacial Muscles During Wear

**DOI:** 10.7759/cureus.59228

**Published:** 2024-04-28

**Authors:** Keiko Kujirai, Masahiro Takahashi, So Koizumi, Kazuhide Seimiya, Toshihumi Nakashizu, Mayumi Watanabe, Tetsutaro Yamaguchi

**Affiliations:** 1 Department of Orthodontics, School of Dentistry, Kanagawa Dental University, Yokosuka, JPN; 2 Division of Dental Practice Support, Department of Dental Technology, School of Dentistry, Kanagawa Dental University, Yokosuka, JPN; 3 Division of the Dental Practice Support, Department of Oral Hygiene Maintenance, School of Dentistry, Kanagawa Dental University, Yokosuka, JPN

**Keywords:** digital technology, 3d printer, orofacial muscle therapy, tongue position, orthodontic appliance, orthodontic treatment

## Abstract

Aim

Balanced function of the orofacial muscles is important for normal occlusion and dentition; however, patients with malocclusion often present with myofunctional disorder (MFD). Myofunctional therapy (MFT) has received much attention as a method for reducing MFD. Moreover, prefabricated functional appliances (PFAs) have been developed as a method to eliminate abnormal muscle pressure and guide the tongue into the correct position. However, PFAs have disadvantages, such as poor intraoral retention, limited usage time due to discomfort and poor patient compliance, and changes in the axis of the mandibular anterior teeth. Therefore, this study aimed to develop a new custom-made splint-type orthodontic appliance with CAD/CAM technology. Moreover, we evaluated the characteristics of the appliance and conducted functional tests to determine the effects of the appliance on the orofacial muscles and the discomfort associated with its use.

Materials and methods

Twenty-five volunteers (nine females and 16 males; mean age 28.4 ± 3.4 years) with normal swallowing function were included in the study. Lip-closing strength (LCS), electromyogram during swallowing, oxygen saturation, and pulse rate were measured and compared when the appliance was not worn and when it was worn. In addition, tongue habits were evaluated, and the maximum tongue pressure was measured when the appliance was not worn. The subjects were asked to answer a questionnaire using a numerical rating scale (NRS) regarding discomfort when wearing the appliance. The evaluation items were swallowing difficulty, speaking difficulty, and breathlessness, which were rated on an 11-point scale ranging from 0 to 10. Statistical tests were conducted using IBM SPSS version 28.0.1 (IBM, Armonk, NY, USA) with the Shapiro-Wilk and Levene's test, followed by the Wilcoxon signed rank sum test. The significance level was set at α = 0.05. The measurement error for each measurement item was evaluated using an intraclass correlation coefficient.

Results

A new custom-made splint-type orthodontic appliance was fabricated for each subject. The fit and retention of the appliance in the mouth were good when fitted, and a comparison of the functional test measurements of 25 subjects with and without the appliance showed that the LCS decreased significantly (p<0.05) before and after wearing the appliance. However, no statistically significant differences were found for the other items. The Mann-Whitney U test regarding the effects of sex, previous orthodontic treatment, or MFT, and oral habits did not statistically significantly influence the effects of wearing the device. In the NRS results, “difficulty swallowing” was observed in half of the subjects, “difficulty breathing” was rarely observed, and “difficulty speaking” was observed in all subjects.

Conclusions

A novel custom-made splint-type orthodontic appliance was designed and fabricated using digital workflow and 3D printing technology. This appliance was designed to correct oral habits and was made from a new material classified as a class II medical appliance according to the international harmonized classification.

## Introduction

The stable function of the orofacial muscles, such as the tongue, orbicularis oris, buccal muscles, and masticatory muscles, is important for the formation and maintenance of normal occlusion and dentition. These muscles are directly involved in important oral functions, such as chewing, enunciation, and breathing, and uneven muscle function is known to cause malocclusion and oral dysfunction [1]. Oral myofunctional therapy (MFT) is based on Rogers’ 1918 study of the relationship between orofacial muscle function and dentition, and it is regarded as an important technique to promote stable function of the orofacial muscles and to improve occlusal health and oral function [2]. In the 1960s, Zickefoose et al. developed a training method for use in the clinical setting that has become widely used, especially in orthodontic treatment [3]. However, the MFT is dependent on patient compliance and effort, making it difficult to manage. Therefore, orthodontic appliances have been developed to improve the position of teeth and jaws and normalize oral function by eliminating abnormal muscle pressure around the dentition and guiding the tongue to the correct position when worn in the mouth [4].

Tongue thrust, or low tongue, is a typical myofunctional disorder (MFD) that causes occlusal abnormalities, and the tongue thrust habit has long been corrected by the use of an orthodontic appliance, the tongue crib, along with MFT [5,6]. In addition, some prefabricated functional appliances (PFAs), such as the Yanagisawa Class III shield, Trainer for Kids, AMCOP® SC, and Occlus-o-Guide® have been developed to improve MFD [7-10]. However, compared to custom-made appliances, PFAs have disadvantages such as poor intraoral fit, which may limit the time of use and cause labial tilting of the mandibular anterior teeth [11].

In recent years, the introduction of digital workflow in dentistry and advances in 3D printer technology have made it possible to create custom-made appliances based on CAD/CAM technology [12]. The classification of medical devices is based on domestic guidelines. In the field of dentistry, materials classified as class I, such as surgical guides for dental surgery, which can be placed in the oral cavity for a short period of time (within 24 hours cumulatively), and materials classified as class II, such as temporary dental prostheses, which can be placed in the oral cavity for a medium term, have been developed [13]. The fabrication of conventional orthodontic appliances is often a complicated process that generates large amounts of medical waste. However, CAD/CAM technology overcomes these problems and has the advantage of easy remanufacturing, making it easier to deal with the loss or damage of devices compared to conventional fabrication methods.

Recently, occlusal splints have been laminated using class II materials [14]. However, an orthodontic appliance to assist MFT using class II materials has not yet been fabricated. Therefore, the purpose of this study was to devise and fabricate a new custom-made splint-type orthodontic appliance for individual volunteers using a newly developed class II material (ink for a 3D printer) that can be placed in the oral cavity for a long period of time and to fabricate a new orthodontic appliance to assist MFT. Furthermore, we compared the results of orofacial muscle function tests when the appliance was not worn and when it was worn and evaluated the characteristics of the new custom-made splint-type orthodontic appliance to obtain knowledge for the clinical application of the appliance.

## Materials and methods

Subjects

Twenty-five adult volunteers (16 males, nine females, 24-37 years, mean age 28.4 ± 3.4 years) working at Kanagawa Dental University Hospital were included (Table 1). The subjects included those with MFD, regardless of whether they had undergone previous orthodontic treatment or MFT.

**Table 1:** Characteristics of the 25 subjects SD: standard deviation, MFD: myofunctional disorder

Factors	Figures
Mean age (SD)	28.37 (±3.43)
Sex	16 males and 9 females
Experience with orthodontic treatment	Yes 13, no 12
Experience with oral myofunctional therapy	Yes 5, no 20
MFD (low tongue and tongue protrusion habit)	Yes 12, no 13

Fabrication of custom-made splint-type orthodontic appliances using a 3D printer

**Figure 1:**
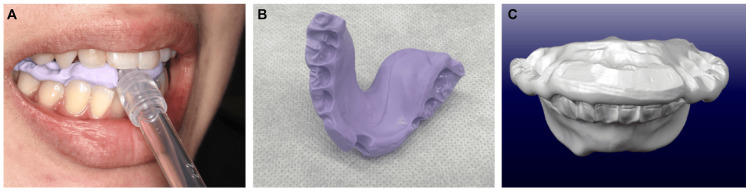
(A) Impression of the tongue during swallowing with a 6.0-mm opening; (B) silicon impression; (C) design using CAD software.

An oral impression was taken with silicon impression material from the lower part of the tongue to the occlusal surface of the dentition during swallowing, with the anterior teeth opened 6.0 mm wide (Figure 1A-1B). An intraoral scanner, iTero® Element™2 (Align Technology, Inc., San Jose, CA, USA), was used to capture the impression and convert it to STL data (Figure 1C). After the appliance was designed, laminate modelling was performed using a 3D printer (Cara® Print 4.0 Pro; Kulzer, Tokyo, Japan) and ink (Dima® Print Mouth Guard; Kulzer).

Functional evaluation

To evaluate the appliance, tongue pressure, LCS, masseter muscle electromyogram (EMG) during swallowing, oxygen saturation (SpO_2_), and pulse rate were measured and compared with and without the appliance in place (Table 2). To evaluate the dynamics of the orbicularis oris muscle when worn, LCS was measured. EMGs of the masseter muscle were measured during swallowing to confirm whether the masseter muscle was hypertonic when wearing the appliance. SpO_2_ and pulse rate were measured to check for breathlessness caused by the appliance.

**Table 2:** Measurement items and types of measuring devices EMG: electromyogram; SpO_2_: oxygen saturation; bpm: beats per minute

Measurement items	Measurement device
Maximum tongue pressure (kPa)	Tongue pressure measuring device (JMS Ltd., Co., Tokyo, Japan)
Lip-closing strength (N)	Lip-strength meter Lipplekun® (Shohu Ltd., Co., Kyoto, Japan)
EMG during swelling (µV)	Myotrac Infiniti, Biograph Infiniti® (Thought Technology, Ltd., Montreal, QC, Canada)
SpO_2_ (%)	Pulse oximeter (KAEI Ltd., Co., Osaka, Japan)
Pulse rate (bpm)	Pulse oximeter (KAEI Ltd., Co., Osaka, Japan)

Measurement of Maximum Tongue Pressure

**Figure 2:**
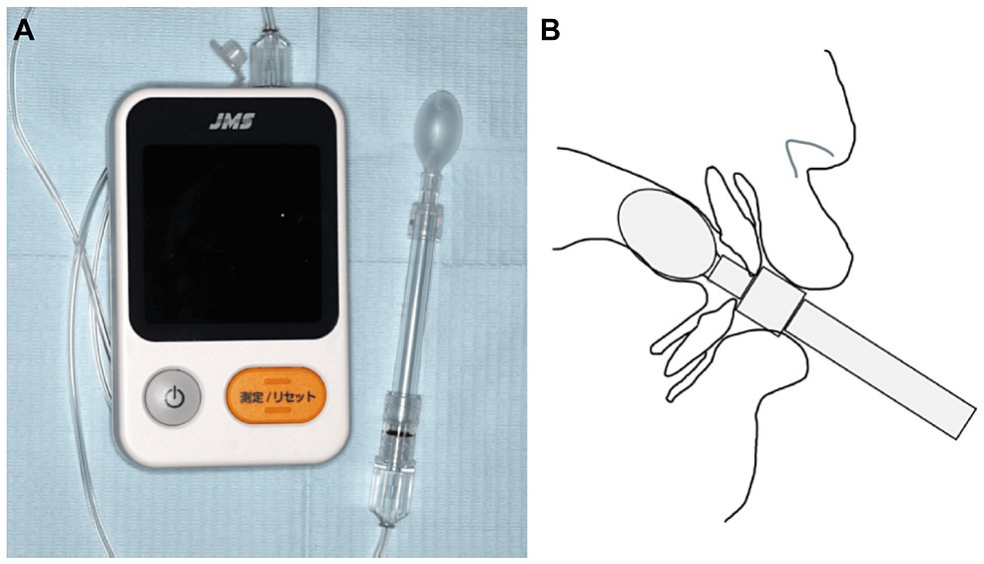
(A) Tongue pressure-measuring device and (B) schematic of the oral cavity during measurement. Image (B) is the self created by the author.

Maximum tongue pressure (MTP) was measured using a tongue pressure-measuring device (JMS Ltd., Co., Tokyo, Japan). Before measurement, the patient was seated upright, and their head was positioned such that the Frankfort horizontal (FH) plane was horizontal to the floor without leaning on a chair. The patient was instructed to deactivate the tongue, insert the probe into the mouth at the default position (the specified length to the convexity of the probe), and apply pressure to the balloon by elevating the tongue (Figure 2). After the first two practice sessions, three measurements were taken repeatedly with a 30-second time interval, and the average value was recorded. To evaluate tongue pressure, the standard deviation of tongue pressure, as reported by Utanohara et al., was used to identify subjects whose measurement was more than one standard deviation below the average value [15].

Measurement of Lip-Closing Strength

**Figure 3:**
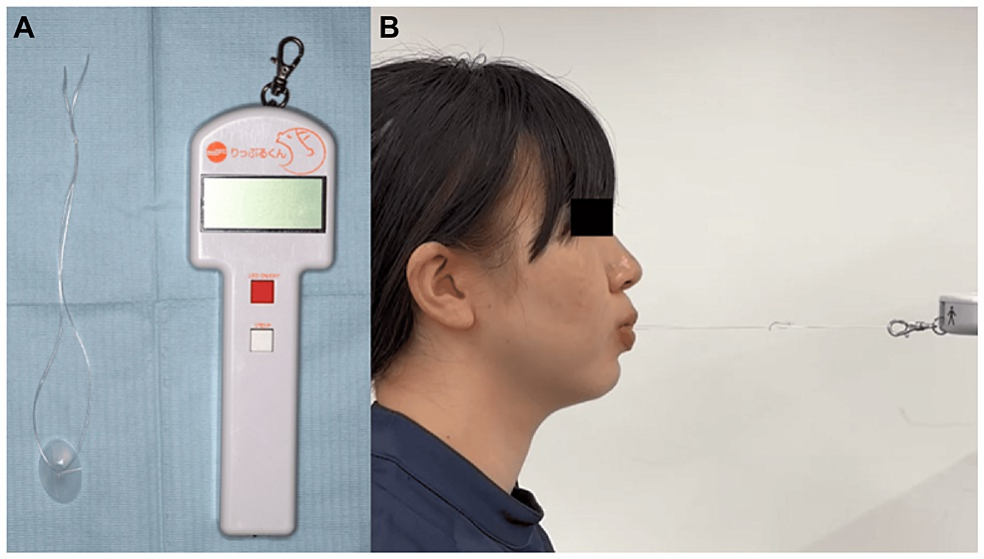
(A) A lip strength meter, Lipplekun; (B) measurement model.

The maximum LCS was measured using a lip-strength meter (Lipplekun®; Shoufu Ltd., Co., Kyoto, Japan). Before the measurement, the subject was placed in an upright position, not leaning against a chair, with their head positioned such that the FH plane was horizontal to the ground, and the patient was instructed to deactivate their lips (Figure 3). A special button was inserted into the oral vestibule, and the button was pulled in a horizontal anterior direction against the face. After the first two practice sessions, the measurements were repeated three times with a 30-second time interval, and the average value was recorded as the measured value and compared in the wearing and non-wearing conditions [16].

Maximum EMG With Swallowing Measurement

Maximum EMG with swallowing was measured using a biofeedback electrical stimulation system (Myotrac Infiniti; Thought Technology, Ltd., Montreal, Canada) and analysis software (Biograph Infiniti®; Thought Technology, Ltd., Montreal, Canada).To record EMGs in the bilateral masseter muscles during swallowing, disposable silver/silver chloride bipolar surfaces (Unigel electrodes; diameter 10 mm) were wiped with ethanol and placed on the muscle belly parallel to the masseter muscle fibers with a distance of 21.0 ± 1.0 mm between electrodes. After the electrodes were applied at the beginning of each subject’s assessment, they remained in position throughout the trial. The subjects were instructed to sit without head support and to maintain a natural upright posture during each trial. Three milliliters of water were placed in the mouth to start the recording, which was repeated three times, recording five seconds from the beginning to the end of the swallow. Muscle activity during swallowing was evaluated as the root mean square of the amplitude (μV), and the mean value of the maximum amplitude was recorded and compared in the wearing and non-wearing conditions [17].

SpO_2_ and Pulse Rate Measurements

SpO_2_ and pulse rate were measured using a pulse oximeter (KAEI Ltd., Co., Osaka, Japan). The measurement environment was a room set at 26 °C. The subjects were seated and instructed to relax by leaning on a chair. The pulse oximeter was placed on the index finger of the left hand, and the values were recorded after three minutes and compared in the wearing and non-wearing conditions.

Evaluation Discomfort During Appliance Wear Using the Numerical Rating Scale

**Figure 4:**
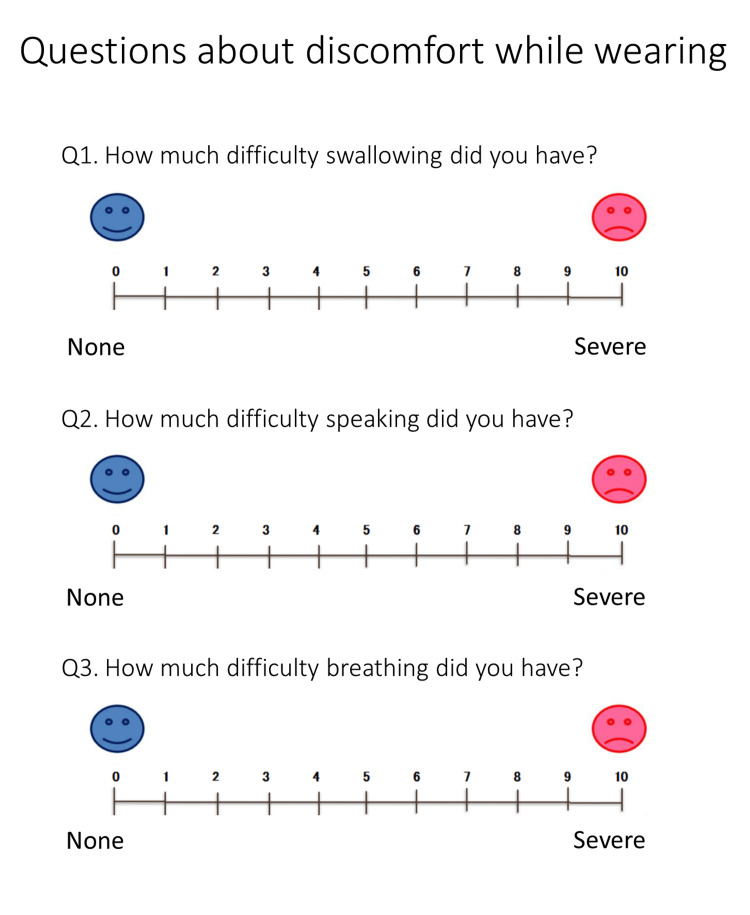
Numerical rating scale. 0: no difficulty in swallowing, speaking, or breathing; 10: major difficulty in swallowing, speaking, or breathing.

Subjects were asked to respond to a questionnaire using an Numerical Rating Scale (NRS) regarding their discomfort when wearing the appliance. The three questionnaire items were difficulty swallowing, speaking, and breathing, which were rated on an 11-point scale from 0 to 10 (Figure 4).

Statistical analysis

Shapiro-Wilk and Levene’s tests were performed on the measurement items using IBM SPSS version 28.0.1 (IBM, Armonk, NY, USA), followed by the Wilcoxon signed rank sum test for the measurement items. The Mann-Whitney U test was used to confirm that each measurement was not affected by subject characteristics (sex differences, previous orthodontic treatment, prior history of MFD, prior MFT experience, and low tongue pressure). The significance level was set at α = 0.05. The measurement error of each measurement item was evaluated by the intraclass correlation coefficient (ICC).

## Results

**Figure 5:**
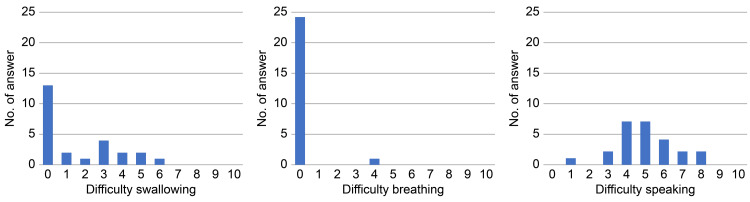
Outcomes of numerical rating scales during the use of the oral appliance.

The ICCs ranged from 0.84 to 0.90, confirming the reliability of each measured item. A custom-made splint-type orthodontic was fabricated for each subject’s unique oral cavity. The geometry of the appliance is illustrated in Figure 5. The anterior part of the maxilla covered the four anterior teeth from the palatal side to prevent protrusion of the tongue and to allow clearance and tooth movement. The posterior tongue-guiding surface was constructed to avoid lingual pedicles and allow the tongue to be guided to the correct position. The area covering the molars was constructed to regulate the vertical movement of the tongue during swallowing. The mandibular dentition was maintained in conformity with the appliance, and the lower-edge morphology of the lingual side of the appliance was determined based on the functional impression of the tongue during swallowing. A good fit was observed in all subjects.

Table 3 compares the results of each functional test in 25 subjects in the wearing and non-wearing conditions. Lip pressure was significantly decreased in the wearing condition, but there were no significant differences for the other parameters.

**Table 3:** Comparison of measured values in the wearing and non-wearing conditions using Wilcoxon signed rank sum test LCS: lip-closing strength; RMM: root mean square of maximal action potential of the right masseter muscle; LMM: root mean square of maximal action potential of the left masseter muscle; Q1: percentile 25; Q3: percentile 75; bpm: beats per minute

Measurements	Wearing			Non-wearing			p-value
	Median	Q1	Q3	Median	Q1	Q3	
LCS (N)	11.20	9.87	13.67	12.83	10.77	15.07	<0.001
RMM (μV)	27.87	20.17	34.21	28.10	22.73	36.56	0.288
LMM (μV)	30.75	24.26	31.79	30.02	27.52	36.15	0.904
SpO_2_ (%)	98.00	97.00	98.00	98.00	97.00	98.00	0.518
Pulse rate (bpm)	66.00	65.00	78.00	70.00	61.00	74.00	0.977

Table 4 shows the Mann-Whitney U test results of the analysis of the influence of various subject characteristics on measurements taken before and after wearing the appliance, including sex (male/female), previous experience of orthodontic treatment, previous experience of MFT, presence or absence of MFD, and MTP (low/normal). There were no statistically significant differences for any of the measured items. Therefore, it was determined that these subject characteristics had no effect on the measurements, and therefore, subjects with various characteristics were treated as one simple group.

**Table 4:** Comparison of difference in subject characteristics by Mann-Whitney U test. LCS: lip-closing strength, RMM: root mean square of maximal action potential of the right masseter muscle, LMM: root mean square of maximal action potential of the left masseter muscle; MFD: myofunctional disorder, MFT: oral myofunctional training, MTP: maximum tongue pressure; Q1: percentile 25; Q3: percentile 75; bpm: beats per minute.

		Male	Female	Not experienced orthodontic treatment	Experienced orthodontic treatment	Not experienced MFT	Experienced MFT	Not have MFD	Have MFD	Low MTP	Normal MTP
	N	16	9	12	13	20	5	13	12	3	22
LCS (N)	Median	0.97	1.10	0.77	1.10	1.10	0.50	1.10	0.77	0.83	1.10
	Q1	0.48	0.50	0.10	0.53	0.58	−0.33	0.63	0.18	0.53	0.46
	Q3	1.92	2.77	1.88	2.20	1.92	2.77	1.37	3.03	2.77	1.77
	P-value	0.890		0.406		0.621		0.936		0.892	
RMM (μV)	Median	5.06	−1.34	6.64	−1.34	2.64	−1.34	1.57	3.57	−1.07	2.64
	Q1	−5.46	−13.76	−1.31	−12.32	−6.77	−15.31	−6.81	−6.83	−12.32	−6.75
	Q3	22.50	5.29	27.04	6.71	13.64	5.29	8.69	17.56	5.29	11.17
	P-value	0.169		0.098		0.488		0.769		0.530	
LMM (μV)	Median	4.88	−2.03	0.55	−1.61	0.18	−2.03	1.12	−1.82	−1.48	−1.19
	Q1	−8.50	−8.41	−10.88	−2.21	−8.50	−8.41	−2.21	−12.35	−8.41	−33.02
	Q3	15.26	−1.48	16.87	1.61	11.69	−1.61	9.67	5.17	1.88	9.76
	P-value	0.108		0.470		0.243		0.295		0.973	
SpO_2_ (%)	Median	0.00	0.00	0.00	0.00	0.00	1.00	0.00	0.50	0.00	0.00
	Q1	0.00	−1.00	−0.50	0.00	−0.50	1.00	0.00	−0.50	−1.00	0.00
	Q3	1.00	1.00	0.50	1.00	0.50	1.00	0.00	1.00	0.00	1.00
	P-value	0.559		0.650		0.169		0.347		0.351	
Pulse rate (bpm)	Median	−0.50	−1.00	1.00	−1.00	0.50	−1.00	−1.00	0.00	4.00	−1.00
	Q1	−4.50	−2.00	−1.50	−5.00	−3.00	−3.00	−4.00	−2.50	−2.00	−4.25
	Q3	3.00	6.00	4.00	3.00	4.00	−1.00	3.00	4.00	4.00	3.00
	P-value	0.329		0.295		0.668		0.650		0.340	

Figure 5 illustrates the outcomes of monitoring discomfort using the NRS during appliance wear. Half of the subjects reported “difficulty swallowing.” Only one subject had “difficulty breathing.” All subjects had “difficulty speaking,” and more than half of them reported an NRS score of five or more.

Based on the LCS results, post-hoc power analysis was performed using G*Power Version 3.1.9.7 (Franz Faul, Kiel University, Kiel, Germany). The statistical power was found to be more than 90%.

## Discussion

Since the achievement of normal occlusion and subsequent retention management in orthodontic treatment also depends on proper orofacial muscle function, it is essential to provide both orthodontic treatment and MFT to correct MFD in patients with MFD. Hassan et al. reported a relationship between orofacial muscles and proper anterior tooth position, and Burstone et al. showed that lower lip muscle strength plays an important role in maintaining proper dental axis inclination of the maxillary anterior teeth at rest [18,19]. In addition, excessive lower lip pressure may be caused by the lingual inclination of the mandibular incisors, mandibular retraction, and class II division 2 malocclusion. Moreover, insufficient strength of the entire orbicularis oris muscle may result in the labial inclination of the maxillary incisors [19]. Rogers found that muscle changes due to functional training of the orofacial muscles affect the positioning of the dental arch, which in turn can lead to changes in dental arch morphology [2]. However, reports suggest that it is difficult to accurately evaluate the effectiveness of MFT because its effectiveness depends on patient compliance, and it is necessary to evaluate the effectiveness of MFT considering the growth and development of the maxillofacial region [20,21]. In addition, studies evaluating the effectiveness of MFT are at a high risk of bias owing to inconsistent protocols and methods in randomized controlled trials, as noted in systematic reviews [20-22]. Thus, scientific evidence supporting the usefulness of MFT for orthodontic treatment remains insufficient.

PFAs are used to normalize orofacial muscle function by removing excess periapical muscle pressure and improving muscle balance at rest. It has been suggested that the combined use of MFT and orthodontic appliances may be more effective in reducing MFD than MFT alone [23]. However, many PFAs are designed to guide and fix the mandible anteriorly, and their time of use may be limited by poor fit. In addition, many disadvantages have been reported, such as labial tilting of the mandibular anterior teeth and changes in lower facial height due to forward retention of the mandible with the teeth [20]. Moreover, studies examining the effect of PFA treatment on MFD have indicated a high risk of bias [20,24].

The Yanagisawa Class III shield is a PFA that negates the need to hold the mandible forward by attaching the appliance to the teeth. It is applied to growing children with functional mandibular prognathism and skeletal mandibular prognathism and is used to balance the orofacial muscles and achieve a proper jaw position. It is designed to stretch the orbicularis oris muscle of the upper lip, promote the forward growth of the maxilla, and guide the tongue to the palate. Onodera et al. reported the effect of elevated tongue position after treatment with the Yanagisawa Class III shield, suggesting that normal jaw position may be achieved by improving tongue function [7]. However, this appliance has the disadvantage of being particularly prone to detachment, which shortens the time it can function in the oral cavity. We considered that to more reliably improve the function of the orofacial muscles when using orthodontic appliances, it is necessary to develop appliances that overcome the drawbacks of PFAs.

Therefore, in the present study, we designed a new custom-made splint-type orthodontic appliance that is expected to have a more significant influence on the orofacial muscles during MFD treatment. The appliance was fabricated using digital workflow and CAD/CAM technology with class II classification materials for long-term retention in the oral cavity. The workflow with CAD/CAM technology also indicated that the orthodontic device could be fabricated with a simpler process compared to the conventional fabrication process. The appliance was designed to improve the fit of the mandibular dentition, not provide anterior mandibular guidance, and was shaped to address the aforementioned drawbacks and minimize the incidence of unfavorable dental changes caused by appliance usage.

Furthermore, to understand the characteristics of the fabricated appliance, a functional test was conducted on the subject for whom the appliance was fabricated and evaluated. The results of the functional test showed that the appliance was stable in the oral cavity, subjects had very little difficulty breathing, and very little abnormal muscle tension was observed when the appliance was worn. The custom-made fabrication not only eliminated the previously reported shortcomings of PFAs and provided excellent retention and fit, but it also showed a significant decrease in LCS. The 6.0-mm occlusal elevation in the anterior teeth was presumed to be the cause, but the appliance did make it difficult to close the lips. Based on the NRS results, difficulty in speaking and swallowing was observed, suggesting that the tongue was compressed by the tongue guide portion. One of the aims of using this appliance is to allow the wearer to develop the habit of maintaining tongue position while keeping the tongue elevated. The above results suggest that it is necessary to adjust the thickness of the tongue guide part and the appliance so as not to impair the effect of tongue elevation [10].

Our study findings align with those of prior research on PFAs, demonstrating a reduction in LCS [23]. However, because this appliance does not have a lip pad that eliminates orbicularis oris pressure, it is possible to directly utilize the orbicularis oris pressure during lip-closing compared to PFAs with a lip pad [25]. Furthermore, as reported by Farronato et al., no significant changes were observed in the EMG of the masseter muscle during swallowing after wearing the appliance, suggesting that the appliance may not induce abnormal clenching or other complications.

Limitations

The new custom-made splint-type orthodontic appliance fabricated in this study to assist MFT was worn by adult volunteers in a preliminary experiment to evaluate the fit, maintenance of the appliance, and condition of the orofacial muscles while wearing the appliance. In future studies, it will be necessary to test the appliance on patients during the mixed dentition period, when growth and development are expected, and to verify the long-term effects of the appliance. To evaluate the effects, it is also necessary to compare the results of patients treated with this appliance with existing treatments. In addition, because it is important to evaluate the stability of the appliance, it is also important to conduct a longitudinal study after treatment. Moreover, there are no studies that have compared the physical properties of this new material. Further studies and reports are needed to examine in more detail the physical properties of the materials used in this appliance and to compare the effects of different materials.

## Conclusions

In this study, CAD/CAM technology was used to fabricate a custom-made splint-type orthodontic appliance to assist MFT. The shape of the appliance consisted of a guiding surface to control tongue movement by preventing tongue thrust, guiding the tongue apex to the correct tongue position, and a maintenance portion to provide wearing stability in the mouth. A comparison of the changes in the functional measures of the orofacial muscles, when the appliance was and was not worn, showed that the appliance was excellent in terms of fit and maintenance, although some modifications in the design of the appliance could be considered.
